# Healthcare resource use and costs of managing children and adults with lysosomal acid lipase deficiency at a tertiary referral centre in the United Kingdom

**DOI:** 10.1371/journal.pone.0191945

**Published:** 2018-02-02

**Authors:** Julian F. Guest, Andy Ingram, Nadia Ayoub, Christian J. Hendriksz, Elaine Murphy, Yusof Rahman, Patrick McKiernan, Helen Mundy, Patrick Deegan

**Affiliations:** 1 Catalyst Health Economics Consultants, Rickmansworth, Hertfordshire, United Kingdom; 2 Faculty of Life Sciences and Medicine, King’s College, London, United Kingdom; 3 Adult Inherited Metabolic Disorders, Salford Royal NHS Foundation Trust, Salford, United Kingdom; 4 Paediatrics and Child Health, University of Pretoria, Pretoria, South Africa; 5 Charles Dent Metabolic Unit, National Hospital for Neurology and Neurosurgery, London, United Kingdom; 6 Adult Inherited Metabolic Disease, Guy's & St Thomas' Hospital, London, United Kingdom; 7 The Liver Unit, Birmingham Children's Hospital, Birmingham, United Kingdom; 8 Evelina Children's Hospital, Guy's & St Thomas' Hospital, London, United Kingdom; 9 Lysosomal Disorders Unit, Addenbrooke's Hospital, Cambridge, United Kingdom; Dartmouth-Hitchcock Medical Center, UNITED STATES

## Abstract

**Objective:**

To estimate clinical progression and resource utilisation together with the associated costs of managing children and adults with LAL Deficiency, at a tertiary referral centre in the UK.

**Methods:**

A retrospective chart review was undertaken of patients in the UK with a confirmed diagnosis of LAL Deficiency who were managed at a LAL Deficiency tertiary referral treatment centre. Patients’ pathways, treatment patterns, health outcomes and resource use were quantified over differing lengths of time for each patient enabling the NHS cost of patient management in tertiary care to be estimated.

**Results:**

The study population comprised 19 patients of whom 58% were male. Mean age at the time of initial presentation was 15.5 years and the mean age at diagnosis was 18.0 years. 63%, 53% and 42% of patients had hepatomegaly, abnormal lipid storage and splenomegaly at a mean age of presentation of 17.8, 17.1 and 20.9 years, respectively. Over a period of 50 years there were a mean of 48.5 clinician visits and 3.4 hospital admissions per patient. The mean NHS cost of patient management at a LAL Deficiency tertiary referral treatment centre, spanning a period of over 50 years was £61,454 per patient.

**Conclusion:**

This study provides important insights into a number of aspects of the disease that are difficult to ascertain from published case reports. Additionally, it provides the best estimate available of NHS resource use and costs with which to inform policy and budgetary decisions pertaining to managing this ultra-orphan disease.

## Introduction

LAL Deficiency (also called cholesterol ester storage disease OMIM 27800) is an autosomal recessive disorder caused by a deficiency of the enzyme, cholesterol ester hydrolase (EC 3.1.1.13). It can manifest as a severe infantile and rapidly progressive disorder, Wolman’s disease, or the more attenuated form which manifests in childhood or adolescence as a progressive disease affecting many organ systems, which is the scope of this article [1,2]. The infantile variant of the disease is characterised by harmful amounts of lipids that accumulate in the liver, spleen, bone marrow, small intestine, adrenal glands, and lymph nodes leaving affected infants suffering from severe malabsorption, failure to thrive, severe liver dysfunction and high rates of mortality. LAL Deficiency in infants has an estimated incidence of between 1 in 350,000 and 1 in 512,000 newborns. These children are estimated to die of the disease at a median age of 3.6 months [3,4].

The less rapidly progressive presentation of LAL Deficiency, often referred to as Cholesteryl Ester Storage Disease and which is the focus of this study, affects both children and adults and has been estimated to affect 3–25 individuals per million worldwide [5]. The median age of first symptoms is around 5 years of age, although many patients remain misdiagnosed or undiagnosed, since symptoms and signs overlap with more common hepatic conditions [1,6]. Other patients remain asymptomatic in spite of extensive underlying pathology and the disease, therefore, may go unrecognised and be identified only after it becomes symptomatic at a very advanced stage [5].

At the time of performing this study, there were 23 known living patients in the UK who were being managed at one of six tertiary referral treatment centres. There was also one known deceased patient who died of a cause unrelated to his/her disease. Based on the incidence estimated by gene frequency [5], there may be at least 200 patients with LAL Deficiency in the UK, implying there are many undiagnosed patients in the UK, although no formal studies have been performed to determine actual prevalence. Some undiagnosed patients will have raised cholesterol concentrations that are undoubtedly being treated with statins. Additionally, there will be other, more advanced, undiagnosed patients who are being treated by gastroenterologists, lipidologists and hepatologists who do not have an index of suspicion for LAL Deficiency. To date, any deceased patients with LAL Deficiency in the UK (except for the aforementioned patient) have died without having been diagnosed.

Documented clinical consequences of LAL Deficiency in patients around the world include hepatomegaly, splenomegaly, liver fibrosis, liver cirrhosis, liver transplant, premature atherosclerosis, premature cardiac events and death. There is no published information on the overall burden of managing patients with LAL Deficiency over time, nor are we aware of any rigorous non-case study information that documents the progression of the disease over time.

In November 2013, the UK Government launched a new plan designed to improve the support, treatment and research for the care of people affected by rare diseases. The strategy aims to ensure that people living with a rare disease have the best quality of evidence-based care and treatment that health and social care systems, working with charities and other organisations, researchers and industry, can provide [7]. The aim of this study was to estimate clinical progression and resource utilisation together with the associated costs of managing children and adults with LAL Deficiency, using data retrospectively retrieved from the clinical records and charts of patients at participating specialist treatment centres.

## Methods

### Study design

This was a retrospective chart review of patients in the UK with a confirmed diagnosis of LAL Deficiency. Infants with early onset Wolman’s disease were beyond the scope of this study and were therefore excluded from the chart review.

### Participating centres

A search was undertaken across the UK to identify centres that were managing patients with a known diagnosis of LAL Deficiency. Six tertiary referral treatment centres were identified. The chart review was performed at five of these six centres. The five centres were:

- Lysosomal Disorders Unit, Addenbrooke's Hospital, Cambridge.
- The Liver Unit, Birmingham Children's Hospital, Birmingham.
- Adult Inherited Metabolic Disorders, Salford Royal NHS Foundation Trust, Salford.
- Charles Dent Metabolic Unit, National Hospital for Neurology and Neurosurgery, London.
- Adult Inherited Metabolic Disease and Paediatric Metabolic Medicine, Guy's & St Thomas' Hospital, London.

### Ethics approval

Ethics approval to perform this study was obtained from the NRES Committee North East—Tyne & Wear South (reference no: 14/NE/1186). Local Research & Development approval was subsequently obtained from each centre, and where required, informed patient consent was also obtained. Due to the small sample size, a condition of the ethics approval was that the level of granularity of data analysis was limited to avoid individual patient identification. This stipulation restricted our ability to report patient-level narratives of disease progression.

### Study population

At the time of performing the chart review there were 24 known paediatric and adult patients with LAL Deficiency in the UK who were managed at one of six centres. All of the known patients were invited to participate in the study. Of these, 5 patients did not provide informed consent. Hence, the chart review was conducted on 19 of the known 24 patients (Table 1).

**Table 1:** Sample size.

Centre	Number of Patients with LAL Deficiency at the centre	Number of LAL Deficiency patients who participated in the study
Addenbrooke's Hospital, Cambridge	7*	7*
Birmingham Children's Hospital	5	5
Salford Royal NHS Foundation Trust	6**	2
National Hospital for Neurology and Neurosurgery, London	3*	3*
Guy's & St Thomas' Hospital, London	3	3
Centre for Liver Research, Birmingham	1***	0
TOTAL NUMBER OF INDIVIDUAL PATIENTS	24	19

* 1 patient was managed at both Addenbrooke’s Hospital and the National Hospital for Neurology and Neurosurgery, London.

** 4 patients did not provide informed consent.

*** 1 patient did not provide informed consent.

### Data collection

The chart review was conducted during 2015. At one hospital, the chart review was undertaken by a specialist nurse employed at the centre. At the other hospitals, the chart review was undertaken by the same health economist (AI) who visited each centre and was given access to patients’ records/charts.

The composition of patients’ records/charts that needed to be reviewed varied between centres. In summary, they included:

- Outpatient notes from each specialty within a multi-disciplinary team.
- Inpatient notes.
- Surgical notes.
- Prescribing charts.
- Diagnostic and laboratory test results.
- Therapeutic procedures.
- Referral letters.

Clinical outcomes and resource utilisation data obtained from patients' records/charts were transcribed onto an electronic database which was purpose-designed for this study. Aggregate data on clinical progression was recorded and reported as (1) rates of resource utilisation, (2) specific complications (such as vascular events), (3) specific interventions for progressive disease (liver transplant), (4) medication use and (5) changes in blood test results.

A unique number was recorded for each patient for tracing original patient records, but the identity code was held only by the clinical investigator at each centre, thereby ensuring patient anonymity. Data were not obtained from patients' records/charts for any period during which they participated in a clinical trial.

### Study variables

Information was systematically extracted from patients’ records/charts in accordance with the protocol. This was achieved by reviewing a range of paper and electronic records/charts at each centre.

The following information was collected during review of the above:

- Age and method of diagnosis.
- Age at symptom presentation.
- Morbidities and treatment strategy for each morbidity.
- Symptoms and clinical outcomes.
- Healthcare resource use (e.g. outpatient care, inpatient care, surgical procedures, diagnostic tests and procedures, laboratory tests, clinical investigations, prescribed drugs).

### Data analysis

Each patient had a different length of history and therefore contributed a different number of years of follow-up data to the study. Consequently, the total value for each variable over a ten year period was calculated for each patient. A mean of all the patients that contributed to each of the ten year periods was then estimated in order to derive meaningful estimates with the minimum of uncertainty. Accordingly, patients’ pathways, treatment patterns, health outcomes and resource use were quantified in the following time segments corresponding with the patients’ age:

- Pre-diagnosis group.
- Post-diagnosis groups: age deciles from 1 to 50 years old.

### Cost of care

Standard NHS reference costs at 2014/15 prices [8,9] were assigned to the healthcare resource use values obtained in the chart review in order to estimate the cost of managing patients with LAL Deficiency in the following time segments corresponding with the patients’ age:

- Pre-diagnosis group.
- Post-diagnosis groups: age deciles from 1 to 50 years old.

The proportional cost attributable to each resource in the management of this disease and the key cost drivers were also characterised. The analysis was conducted from the perspective of the UK’s NHS.

Additionally, patients’ resource use was individually costed for each year they had data and used to create a statistical mixed model, containing both fixed effects and random effects. The output from this model (i.e. area under the curve) is an estimate of the mean cost of managing a patient over time. Accordingly, the mixed model estimated the mean cost of managing an LAL Deficiency patient at a tertiary referral treatment centre over 50 years. An advantage of mixed models is that they naturally handle uneven spacing of repeated measurements, whether intentional or unintentional. Additionally, mixed model analysis can incorporate non-normally distributed dependent variables.

There was some variation between the standard NHS reference costs and the tariffs that may have been agreed at a local level, particularly for surgical procedures. The impact of such variance was tested with sensitivity analysis.

### Sensitivity analyses

To assess whether any variable had a major impact on the total cost of patient management, one-way sensitivity analyses were performed on resource use values collected during the chart review and unit costs. Accordingly, resource use values and unit costs were decreased and increased by 25% to assess how this affected the total cost of patient management. A scenario was also created to assess the cost impact of every patient undergoing a liver transplant.

## Results

### Age of clinical presentation and diagnosis

The sample of 19 patients provided a total of 343 patient years of data. However, the follow-up time for the sample was heterogeneous. One third of the cohort had a total of 36 patient years, whereas another third had a total of 200 patient years. For each age decile:

- 1–10 years of age post-diagnosis, 53% of the cohort contributed data.
- 11–20 years of age post-diagnosis, 47% of the cohort contributed data.
- 21–30 years of age post-diagnosis, 47% of the cohort contributed data.
- 31–40 years of age post-diagnosis, 42% of the cohort contributed data.
- 41–50 years of age post-diagnosis, 42% of the cohort contributed data.

Additionally, some of these patients were enrolled in a clinical trial, during which time the study did not capture their resource utilization. Therefore, for these patients there is a discontinuity in the capture of their data.

Fifty-eight percent of the patients were male. The mean age at the time of presentation of the first symptom was 15.5±14.6 years and the mean age at diagnosis was 18.0±14.2 years. Enzyme tests were used to make a diagnosis in 95% of patients and DNA analysis was undertaken in 47% of patients. At least half the patients underwent multiple methods of diagnosis. The method of diagnosis and the clinician who made the diagnosis is summarised in Table 2, and reflects the way these patients were diagnosed in clinical practice.

**Table 2:** Patients’ characteristics.

	Percentage of patients
Method of diagnosis*	
Enzyme tests	95%
Abnormal lipid storage	68%
DNA analysis	47%
Family screen	11%
**Clinician who made the diagnosis**	
Metabolic consultant	32%
Paediatric hepatologist	26%
Paediatrician	16%
Unknown	16%
Other physician	10%

*Patients underwent more than one method of diagnosis.

Patients started experiencing different disease-related signs, symptoms and features at a mean age of 17 years, although bruising started at a mean age of 13 years. Sixty-three percent of patients presented with hepatomegaly at a mean age of 17.8 years, 53% were found to have abnormal lipid storage (as determined by biopsy and/or ultrasound) at a mean age of 17.1 years and 42% presented with splenomegaly at a mean age of 20.9 years. Patients presented with a mean of 3.2 different signs, symptoms and features and 95% of the patients presented with >1 sign, symptom or feature. The percentage of patients who experienced different signs, symptoms and features and the corresponding mean age at initial presentation is shown in Table 3.

**Table 3:** Patients’ disease-related signs, symptoms and features.

	Percentage of patients:		
	Pre-diagnosis	Post-diagnosis	Mean age at initial presentation (years)
Hepatomegaly	63%	74%	17.8
Abnormal lipid storage	53%	58%	17.1
Splenomegaly	42%	74%	20.9
Hepatic fibrosis	37%	53%	17.6
Abdominal/epigastric pain	32%	58%	24.2
Hepatic dysfunction	26%	37%	25.4
Diarrhoea	26%	37%	21.2
Portal hypertension	26%	53%	22.5
Lethargy/fatigue	21%	42%	24.7
Vomiting	21%	26%	17.0
Neurological symptoms	21%	47%	24.6
Abnormal lipid profile	16%	68%	23.2
Respiratory symptoms	16%	42%	19.0
Spider naevi	11%	26%	23.3
Bruising	11%	16%	12.8
Weight loss	5%	16%	31.5
Weight gain	5%	21%	35.8
Stenosis of blood vessels	0%	21%	27.3

### Clinician visits

**Fig 1:**
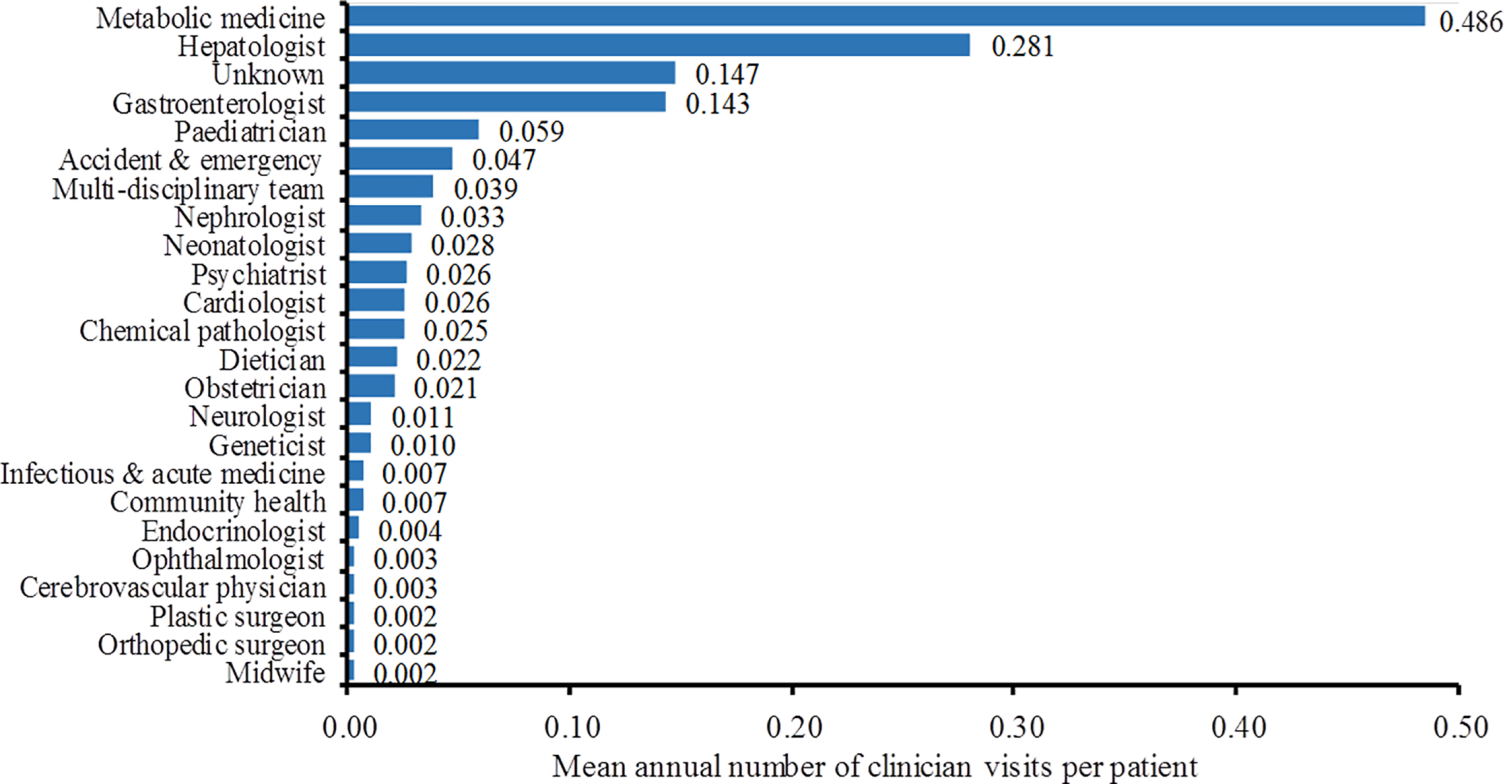
Mean annual number of post-diagnosis clinician visits per patient.

Patients had a mean of 48.5±14.4 clinician visits over a period of 50 years, equating to a mean of 1 visit per year. However, this varied according to age and clinician type. Fig 1 shows the array of clinicians managing patients with LAL Deficiency at a tertiary referral treatment centre and the mean annual number of clinician visits per patient. Patients aged between 1 and 10 years had a mean of 8 clinician visits during that period doubling to a mean of 16 clinician visits between the ages of 11 and 20 years. The number of clinician visits was reduced by 75% in the next decade, and then increased by a mean of 4 visits for each subsequent decade up to 41–50 years of age (Table 4).

**Table 4:** Healthcare resource use.

	Mean amount of resource use per patient					
	Pre-Diagnosis	Age decile post-diagnosis				
Resource		1–10	11–20	21–30	31–40	41–50
Clinician visits	8.20	8.00	16.43	4.00	7.50	12.56
Hospital admissions	0.47	0.45	0.22	0.33	1.01	1.38
Surgical procedures						
Adenotonsillectomy	0.00	0.09	0.00	0.00	0.00	0.00
Cervical loop excision	0.00	0.00	0.00	0.11	0.13	0.00
Chest drain	0.00	0.00	0.00	0.00	0.00	0.88
Cholecystectomy	0.00	0.09	0.11	0.00	0.00	0.13
Emergency caesarean	0.00	0.00	0.00	0.00	0.13	0.00
Liver transplant	0.00	0.09	0.00	0.00	0.00	0.38
Manipulation of joint under anaesthesia	0.05	0.00	0.00	0.00	0.00	0.00
Minor skin procedure	0.11	0.00	0.00	0.11	0.38	0.00
Resection and anastomosis of small intestine	0.05	0.00	0.00	0.00	0.00	0.00
Salpingectomy	0.05	0.00	0.00	0.00	0.00	0.00
Splenectomy	0.05	0.00	0.00	0.00	0.00	0.00
Termination of pregnancy	0.00	0.00	0.00	0.11	0.00	0.00
Clinical Investigations						
Angiogram	0.00	0.00	0.11	0.00	0.00	0.00
Biopsy	0.63	0.36	0.67	0.22	0.13	0.63
Cardiopulmonary exercise test	0.00	0.00	0.00	0.00	0.00	0.38
Colposcopy	0.00	0.00	0.00	0.44	0.13	0.00
CT scan	0.11	0.00	0.00	0.00	0.00	1.00
Dexa scan	0.00	0.00	0.11	0.00	0.00	0.13
Doppler/ultrasound	0.42	0.91	3.00	0.11	0.50	3.63
ECG	0.11	0.27	1.00	0.00	0.13	0.88
EEG	0.11	0.09	0.00	0.00	0.00	0.00
Endoscopy	0.26	0.27	0.67	0.00	0.13	0.25
Histology	0.05	0.00	0.00	0.00	0.13	0.13
MRI	0.26	0.09	0.44	0.00	0.00	0.88
Nuclear medicine	0.05	0.09	0.11	0.00	0.00	0.00
Transient elastography	0.00	0.00	0.00	0.11	0.00	0.00
X-ray	0.21	0.64	0.33	0.00	0.00	1.50
Laboratory tests						
Disease screen	3.89	0.18	1.44	0.11	0.13	4.38
Haematology	46.11	18.00	29.22	3.22	5.75	137.38
Immunology	1.63	0.27	2.00	0.00	0.00	1.88
Lipid pattern	5.63	3.73	12.56	1.89	8.75	37.38
Liver function	17.95	18.09	28.11	4.00	7.75	63.75
Other biochemistry	20.37	18.00	29.33	1.56	7.38	108.38

### Treatments and interventions

Patients had a mean of 3.4±1.9 hospital admissions over a period of 50 years, ranging from 0.5±0.5 admissions per patient between 1–10 years of age, rising to 1.4±3.1 admissions per patient between 41–50 years of age (Table 4). The mean length of hospital stay was 6.5±14.4 days. Seventy-eight percent of admissions were elective, and the other 22% were emergency admissions. Sixteen percent of patients had undergone a liver transplant. Over the study period, patients had a mean of 3.0±2.6 surgical procedures. Attribution of causation of surgery is beyond the scope of this study. The mean number of surgical procedures in each period is summarised in Table 4.

Patients had a mean of 20.6±11.2 clinical investigations and 554.6±359.5 laboratory tests over the study period of 50 years. The number of clinical investigations and laboratory tests stratified by each period is shown in Table 4.

Forty-two percent of all patients were prescribed medication before their diagnosis of LAL Deficiency and 95% of all patients were prescribed medication after their diagnosis. It is noteworthy that pre-diagnosis, 16% of patients were prescribed lipid-lowering medication (statins) and post-diagnosis that increased to 84%. There was a similar increase in prescribed clinical nutrition (comprising enteral and parenteral vitamins B1, B3, B9, C, D, K, iron, calcium, garlic, linseed oil, multivitamins and potassium salts) post-diagnosis (Table 5). Twenty-one percent of patients were prescribed clinical nutrition pre-diagnosis and 37% post-diagnosis.

**Table 5:** Patients who received prescriptions for drugs and clinical nutrition.

	Percentage of patients who received prescriptions	
	Pre-diagnosis	Post-diagnosis
Cardiovascular drugs	16%	84%
Gastroenterologicals	21%	53%
Clinical nutrition	21%	37%
Anti-infectives	16%	32%
Analgesics	16%	26%
Immunologicals	21%	26%
Neurologicals	5%	21%
Anti-poisons	5%	5%
Cytotoxins	0%	5%
Dermatologicals	0%	5%

**Fig 2:**
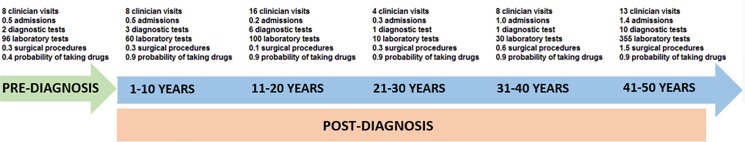
Patient algorithm.

The estimates of healthcare resource use, stratified by time, enabled an algorithm for the 19 patients in the data set to be mapped out (Fig 2).

### Laboratory test results

The results of selected laboratory tests are shown in Table 5. Alanine aminotransferase and aspartate aminotransferase were both elevated in patients <16 years of age, but then levels declined as patients became older. Gamma-glutamyl transferase levels were elevated in patients >10 years of age and remained elevated until patients were >25 years of age. Low density lipoprotein and total cholesterol were both elevated in patients <26 years of age, declining as patients got older, reflecting increased use of statins. Conversely, there was a marked increase in bilirubin levels in patients >20 years of age. The results of the other tests remained unchanged over the study period. Notwithstanding this, evaluating whether the results of the tests were within a target range is beyond the remit of this study (Table 6).

**Table 6:** Laboratory test results.

			Mean value over a five year period post-diagnosis									
Laboratory test	Units	Mean value pre-diagnosis	0–5 years	6–10 years	11–15 years	16–20 years	21–25 years	26–30 years	31–35 years	36–40 years	41–45 years	46–50 years
Albumin	g/L	37.96	42.38	42.75	N/A	46.03	N/A	N/A	44.50	41.29	43.61	44.12
Alanine aminotransferase	IU/L	76.45	94.50	110.10	100.11	78.75	66.10	39.00	88.25	60.53	67.94	31.69
Aspartate aminotransferase	IU/L	167.44	87.75	119.12	71.93	50.38	N/A	40.00	N/A	N/A	N/A	35.00
Gamma-glutamyl transferase	IU/L	48.86	24.50	42.38	80.04	74.17	127.50	41.50	26.00	29.60	79.83	32.00
High density lipoprotein	mmol/L	0.88	0.60	0.80	0.81	0.93	0.90	1.25	0.70	0.73	0.88	0.88
Low density lipoprotein	mmol/L	4.27	6.35	N/A	5.10	4.50	7.20	3.55	3.90	5.31	2.80	2.63
Total cholesterol	mmol/L	5.89	7.58	5.99	5.78	5.49	9.40	5.10	6.05	7.16	4.45	4.19
Triglycerides	mmol/L	1.98	2.25	2.71	2.53	2.34	2.80	1.45	2.70	2.48	1.79	1.99
Activated partial thromboplastin time	Seconds	29.71	34.00	33.42	32.28	32.10	N/A	N/A	N/A	29.40	31.21	33.00
Prothrombin time	Seconds	14.79	11.00	14.55	11.39	10.80	N/A	N/A	N/A	13.25	12.91	11.10
Bilirubin	μmol/L	13.52	7.50	8.71	13.83	12.38	25.15	16.25	56.75	22.93	23.14	24.90
HDL:LDL ratio		8.12	8.06	8.12	8.15	5.60	3.36	3.80	10.10	10.22	6.10	3.97

N/A denotes no values available for those periods.

### Cost of patient management

The mean NHS cost of diagnosis and managing patients post-diagnosis at a tertiary referral treatment centre is shown in Table 7. Prescribed medication accounted for 56% and 46% of the post-diagnosis management cost in the 21–30 and 31–40 age deciles respectively. However, hospitalisation accounted for 56% of the post-diagnosis management cost in the 41–50 age decile. In the 1–10 and 11–20 age deciles, clinician visits were a cost driver accounting for 31% and 44% of the post-diagnosis management cost, respectively (Table 7). The costs reflected a bimodal distribution of healthcare resource use over the 50 years time-horizon. These costs excluded the cost of patient management both in the community and at secondary care centres. Output from the mixed model estimated that the NHS cost associated with LAL Deficiency diagnosis and management over 50 years was a mean of £61,454±1,152 per patient.

**Table 7:** NHS cost of patient management at 2014/15 prices.

	Mean cost of resource use per patient					
	Pre-Diagnosis	Age decile post-diagnosis				
Resource		1–10	11–20	21–30	31–40	41–50
Diagnosis	£41.16					
Clinician visits	£1,429.67	£1,868.72	£3,837.56	£934.36	£1,751.93	£2,932.86
Clinical investigations	£303.95	£322.28	£662.67	£103.85	£101.26	£992.26
Hospitalisation	£1,970.40	£1,916.18	£246.38	£559.89	£504.99	£14,352.21
Laboratory tests	£877.89	£707.36	£1,316.44	£182.78	£504.00	£3,493.13
Prescribed medication	£339.12	£1,176.31	£2,645.44	£2,227.84	£2,414.64	£3,965.78
Cardiovasculars	£9.59	£347.91	£1,296.98	£1,073.00	£1,061.00	£1,049.99
Clinical nutrition	£156.42	£682.41	£877.98	£833.69	£919.27	£947.73
Immunologicals	£85.97	£11.17	£48.60	£45.82	£29.89	£879.64
Dermatologicals	£0.00	£0.00	£172.00	£158.93	£178.80	£178.80
Gastroenterologicals	£47.18	£114.95	£158.80	£90.29	£121.40	£61.58
Neurologicals	£5.08	£19.85	£24.27	£24.39	£27.97	£486.63
Anti-infectives	£2.95	£0.00	£1.01	£0.15	£0.12	£285.06
Analgesics	£31.81	£0.00	£65.81	£0.13	£76.19	£76.35
Cytotoxins	£0.00	£0.00	£0.00	£1.19	£0.00	£0.00
Anti-poisons	£0.12	£0.00	£0.00	£0.25	£0.00	£0.00
Gynaecologicals	£0.00	£0.00	£0.00	£0.00	£0.00	£0.00
**TOTAL**	**£4,962.19**	**£5,990.85**	**£8,708.49**	**£4,008.72**	**£5,276.82**	**£25,736.24**

The total cost of prescribed medication amounted to a mean of £12,769 per patient (Table 7). Of this, cardiovascular drugs accounted for 38% and clinical nutrition for 34%. The other drug classes each accounted for <10% of the total cost of prescribed medication.

### Sensitivity analyses

Sensitivity analysis examined the effect of variability and uncertainty surrounding the information collected during the chart review and demonstrate that our estimate of the NHS cost of patient management over 50 years was relatively robust to changes in resource use values. If the number of:

- Clinician visits was changed by +/- 25%, the NHS cost of patient management over 50 years would change by +/- 1% per patient.
- Hospital admissions and surgical procedures was changed by +/- 25%, the NHS cost of patient management over 50 years would change by +/- 9% per patient.
- Prescriptions was changed by +/- 25%, the NHS cost of patient management over 50 years would change by +/- 6% per patient.
- Laboratory tests was changed by +/- 25%, the NHS cost of patient management over 50 years would change by +/- 3% per patient.
- Clinical investigations was changed by +/- 25%, the NHS cost of patient management over 50 years would change by +/- 1% per patient.

Sensitivity analysis also showed that if every patient had a liver transplant, the NHS cost of patient management over 50 years would increase by 28% to a mean of £70,197 per patient.

If the cost of surgical procedures was changed by +/- 25%, the NHS cost of patient management over 50 years would change by +/- 8% per patient.

## Discussion

To the authors’ knowledge, this was the first study to assess the clinical progression, healthcare resource utilisation and associated costs of managing children and adults with LAL Deficiency in the UK over the course of 50 years. The findings from the study were based on diagnosed patients who had been referred to a specialist treatment centre. Hence, the health economic burden imposed by the disease among diagnosed patents who had not been referred to a specialist treatment centre and undiagnosed patients remains a study limitation. Nevertheless, the analysis characterises the clinical features of 79% of the known cohort of patients with the disease who were managed at a specialist treatment centre in the UK, and provides insights into the impact of current disease management approaches. There is an under-recognition of LAL Deficiency and it is frequently misdiagnosed or confused with other more common liver diseases. Hence, the sample size of this study was small in absolute terms, but it does reflect 79% of the known patient cohort with this ultra-orphan disease in the UK.

It has been reported that patients with LAL Deficiency experience a substantial disease burden, in terms of liver fibrosis and cirrhosis, at <10 years of age [10]. This study supports the findings of others that LAL Deficiency primarily targets the liver, causing hepatic dysfunction. However, the mean age of presentation of different symptoms ranged between late teens and early twenties, which was older than that reported in other populations with this disease [6,10,11]. This difference may be due to the UK having a genuinely milder population, or the search strategy in the US studies [10,11] may have failed to select adults with milder disease. Nevertheless, the features of LAL Deficiency have the potential to lead to significant morbidity and early mortality. Furthermore, a decrease in transaminases over time/disease progression may be interpreted as a manifestation of progressive liver disease, although there is limited evidence of this in our cohort of study patients. Liver transplants are, by the very nature of the entry criteria for listing for transplantation, an indicator that patients would have died of the condition had they not been transplanted.

The study design was primarily aimed at gathering and analysing resource utilisation data. As we were restricted by conditions of the ethics approval not to report patient-level narratives, we anticipated that any evidence of clinical progression would emerge in the aggregate data. However, there was little evidence of gradual progression in most patients, but severe deterioration to the point of requiring liver transplantation in a subset of 16% of the cohort. This fits with our general clinical impression that patients with LAL Deficiency often appear well, until they deteriorate rapidly to decompensated liver disease. In other words, deterioration is either non-existent or subclinical in most patients for long periods of time, and then it becomes rapid in a subset of patients. Slow, steady, clinically evident deterioration is the exception.

In practice, the concerns of patients and medical professionals did not prompt a particularly high use of healthcare resources, since the frequency of visits to a hospital physician at a tertiary referral centre ranged from one visit every two years to two visits every three years, depending on age. Additionally, these patients had a mean of three hospital admissions over a period of 50 years. Notwithstanding this, patients’ use of healthcare resources seemed to increase between the ages of 11 and 20 years, which may be a consequence of an onset of disease-related abnormalities as well as changes within their personal circumstances, such as adolescence, moving from primary to secondary school, leaving secondary education and either going on to further education or starting employment, and starting relationships. Patients’ use of healthcare resources seemed to decline in the third decade but increased markedly between the ages of 41 and 50 years. This may also reflect the natural history of the disease, passing through a symptomatic childhood phase followed by a clinically quiescent early adult phase, during which silent progression of chronic liver disease leads to the presentation of clinical features of liver dysfunction, consistent with the data reviewed by Bernstein and coworkers [6]. Accordingly, their management at a tertiary referral centre was estimated to cost the NHS approximately £61,500 per patient over 50 years, equivalent to £1,200 per patient per year. Furthermore, it seems likely that at different times in the patients’ lives, LAL Deficiency has the potential to impact on their future productivity and ensuing societal costs. Nevertheless, this information was not documented in the patients’ records/charts. Therefore, an estimation of the impact of the disease on indirect societal costs would have required too many assumptions and subjected the analysis to a high degree of uncertainty.

In a recent study in which the authors estimated the burden of wounds in the UK [12], we also estimated the NHS cost of managing members of the general public >18 years of age. The analysis showed that the healthcare cost of managing individuals over the age deciles of 21–30 years, 31–40 years and 41–50 years was a mean of £520, £1,300 and £1,400 per individual, respectively. This indicates that while the patients with LAL Deficiency in this study did not appear to utilise many healthcare resources, they did in fact use substantially more than members of the general public. Notwithstanding this, the cost of managing LAL Deficiency at a tertiary referral centre appears to be less than that of managing some other orphan diseases. The undiscounted lifetime cost (at 2009 prices) in the Netherlands of managing (1) untreated Fabry disease which has a prevalence of around 1 in 40,000 was estimated to be €270,964 (equivalent to £210,000) per patient at a specialist centre [13] and (2) untreated type 1 Gaucher disease which has a prevalence of around 1 in 70,000 was estimated to be €171,780 (equivalent to £133,000) per patient at a tertiary referral centre [14]. We were unable to find any other publications detailing costs associated with managing LAL Deficiency nor any other studies describing the lifetime costs of managing other liver diseases in European countries with a comparable publicly-funded healthcare system to that of the UK. However, the cost of usual care for managing a 50 year old patient with non-alcoholic fatty liver disease over 30 years in the US was estimated to be $98,815 (equivalent to £68,000) per patient at 2014 prices [15].

The advantage of using data from a retrospective chart review is that the patient pathways and associated resource use are based on real world evidence derived from clinical practice. Nevertheless, the possibility of resource use associated with managing LAL Deficiency being conflated with a comorbidity cannot be excluded. Additionally, the possibility of resource use dictated by a clinical trial protocol being conflated with that of usual clinical practice cannot be completely excluded. While the study results are compelling, the analyses were based on clinicians’ entries into their patients’ records and inevitably subject to a certain amount of imprecision and variation in detail. Furthermore, there may be gaps in historical data prior to diagnosis, particularly in adults, as the information may not have been deemed relevant at the time of referral or may not have been fully transferred either from paediatric units or from secondary care centres to the tertiary referral centres that participated in this study. Prescriptions issued by the clinicians were noted in the records, but it does not specify whether the prescriptions were dispensed or detail patient compliance with the medication. It is also noteworthy that while drug prescribing may have been initiated by a physician at the tertiary referral centre, many of the repeat prescriptions would have been written by general practitioners and dispensed in the community. Again, only care provided through the tertiary referral centres is captured in this study. Furthermore, the genotype in the patients in this data set was not available. Despite these limitations, it is the authors’ opinion that this chart review provides a benchmark for understanding how LAL Deficiency is managed within the tertiary care system in the UK.

The analysis does not consider the potential impact of managing patients beyond 50 years of age, and indeed 58% of the patients in the cohort had not reached >40 years of age at the time of performing the chart review. No assumptions were made regarding missing data and there were no interpolations. The analysis may have under-estimated use of some healthcare resources if they were not documented in the patients’ records, but the impact of this was addressed in the sensitivity analyses. Additionally, the analysis excluded community-based management by general practitioners and practice nurses, management at secondary care centres as well as the potential impact of patient management by community nurses. Consequently, the human resource impact as well as the impact on health care resource utilisation of managing patients with LAL Deficiency beyond a tertiary referral centre is unknown. The study also included LAL Deficiency patients who were enrolled in a clinical trial, during which time the study did not capture their resource utilization. Therefore, for these patients, there is a discontinuity of their management and/or treatment captured by the study.

The analysis only considered the annual cost of NHS resource use for the ‘average patient’ and no attempt was made to stratify resource use and costs according to gender, symptoms, disease severity and other disease-related factors, since to do so may have facilitated the identification of individual patients. Also excluded were the costs incurred by patients and indirect costs incurred by society as a result of patients taking time off work. Furthermore, the analysis is presented according to patients’ contribution to each age band and not according to their age at diagnosis. An analysis of the data stratified by patients’ age at diagnosis would be misleading since it ranged from 2 to 43 years (mean of 18.0 ±14.2 years). Furthermore, due to the small sample size, the level of granularity of data analysis was limited in order to avoid individual patient identification. The limitations herein highlight the practical difficulties of performing a chart review among a very small cohort of patients with an extremely rare disease. Furthermore, as a result of these limitations, generalising these findings to other care settings and other healthcare systems becomes very challenging.

### Conclusion

Notwithstanding the study’s limitations, the real world evidence in this study provides the first detailed characterisation of the cohort of patients diagnosed with LAL Deficiency in the UK and provides important insights into a number of aspects of the disease that are difficult to ascertain from published case reports. Additionally, it provides the best estimate available of NHS resource use and costs with which to inform policy and budgetary decisions pertaining to managing this ultra-orphan disease. Clinical and economic benefits could accrue from an increased awareness of LAL Deficiency and improving the differential diagnosis of this disease from other more common causes of chronic liver diseases.

## Abbreviations

LAL: Lysosomal acid lipase
NHS: National Health Service
UK: United Kingdom
